# Supplementary material for: A large decrease in the magnitude of seasonal fluctuations in mortality among elderly explains part of the increase in longevity in Sweden during 20th century
Source: BMC Public Health. 2020 Nov 9;20:1674. doi: 10.1186/s12889-020-09749-4 (PMC7654045; doi:10.1186/s12889-020-09749-4)
Supplement: Supplementary file 1 — Additional file 1 Supporting materials. Contains supporting figures and tables. Standard PDF-format. [file 12889_2020_9749_MOESM1_ESM.pdf]

Supporting material for: A large decrease in the magnitude of seasonal fluctuations in mortality among elderly explains part of the increase in longevity in Sweden during 20th century

Anders Ledberg*

Department of Public Health Sciences, Stockholm University
SE-106 91 Stockholm, Sweden

October 11, 2020

List of Figures

S1 Parameter fits with two different estimators 3
S2 Peak location 4
S3 Mortality rates for two different birth years 5

List of Tables

S1 Size of birth cohorts 7
S2 Survival data for men 8
S3 Survival data for women 9

*anders.ledberg@su.se or anders.ledberg@gmail.com

1 Approximation by Poisson regression

To derive Eq. 2 in the main text a number of approximations were made and here the effect of these approximations are discussed. To measure time in days instead of continuously has no consequences for the age range used in this study; mortality rates change on a much slower time scale.

The expected number of deaths on day t was approximated as

$$E(d_t) = N_t \lambda(t).$$

Note that the correct expression is $E(d_t) = (1 - e^{-\lambda})N$, and the error in approximating $(1 - e^{-\lambda})$ by λ is quadratic in λ by Taylor's theorem. Consequently, this is an excellent approximation when $\lambda(t)$ is small. Recall that $\lambda(t)$ is the mortality rate on day t and note that even at very advanced ages, were the one-year survival probability is some small number, 0.1 say, $\lambda(t)$ will still not be bigger than 0.01. That is, d_t will be well approximated by a Poisson random variable with expected value $N_t \lambda(t)$ throughout.

The final approximation concerns the day of birth for person i, that is b_i in Eq. 1 in the main text. To derive Eq. 2 it was tacitly assumed that all persons belonging to the same cohort were born on the same day, in exactly the middle of the year. This assumption made it possible to use the same mortality rate for all people belonging to the same cohort. Of course, people are born throughout the year, and using Eq. 2 to estimate parameters of Eq. 1 might consequently introduce a small bias in the estimates. Note that for t fixed, Eq. 2 represent the average log mortality rate for those still alive at t, and those born early in the year will have a slight tendency of dying before those born later in the year (due to the age difference), possibly introducing a bias. The magnitude of this bias was investigated by comparing estimates obtained through fitting Eq. 2 to the data using Poisson regression with estimates obtained by a direct maximization of the likelihood corresponding to Eq. 1 and were found to be negligible (see Figure S1). Parameter estimation based on Eq. 2 is preferred because it can be done swiftly using existing routines, and it can moreover be easily extended to incorporate other time-varying factors.

2 Figures

2.1 Parameter estimates

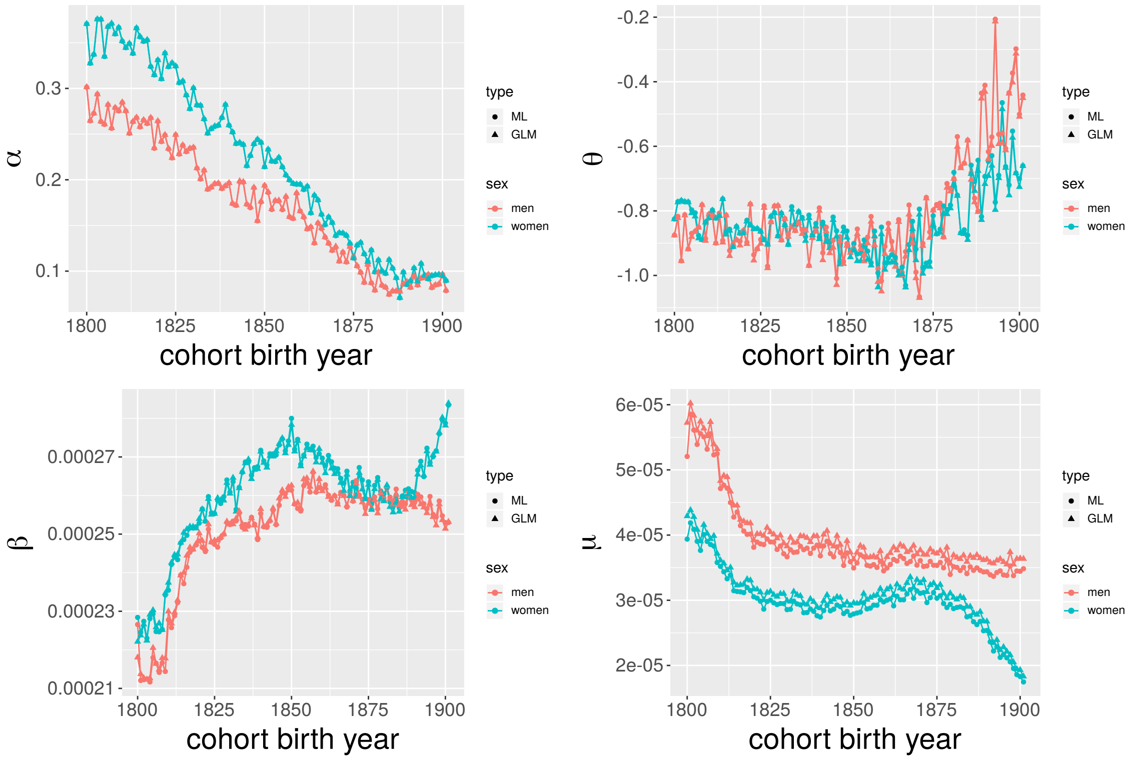

Figure S1: Parameter fits using Poisson regression ("GLM" triangles) and maximum likelihood based on Eq. 1 in the main paper ("ML" circles). The systematic difference in estimates of μ is due to that the estimates based on Eq. 1 are made for someone 59 years of age at time zero, and the Poisson estimates for someone who is 59.5 years.

2.2 Seasonal location of mortality peak

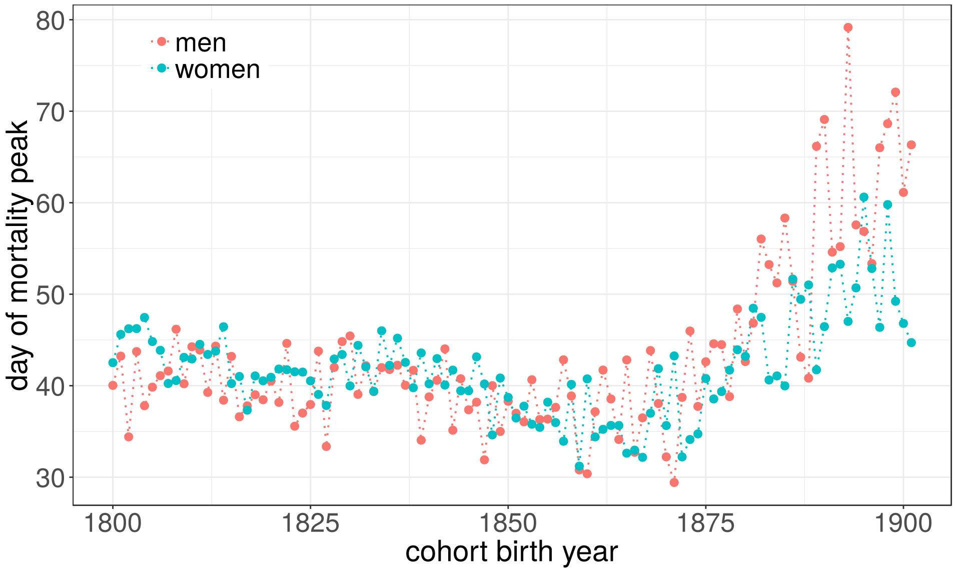

Figure S2: Location of the peak of the seasonal mortality rate fluctuation in days from beginning of the year. The location of the peak was obtained from the model fits.

2.3 Change in the amplitude of seasonality

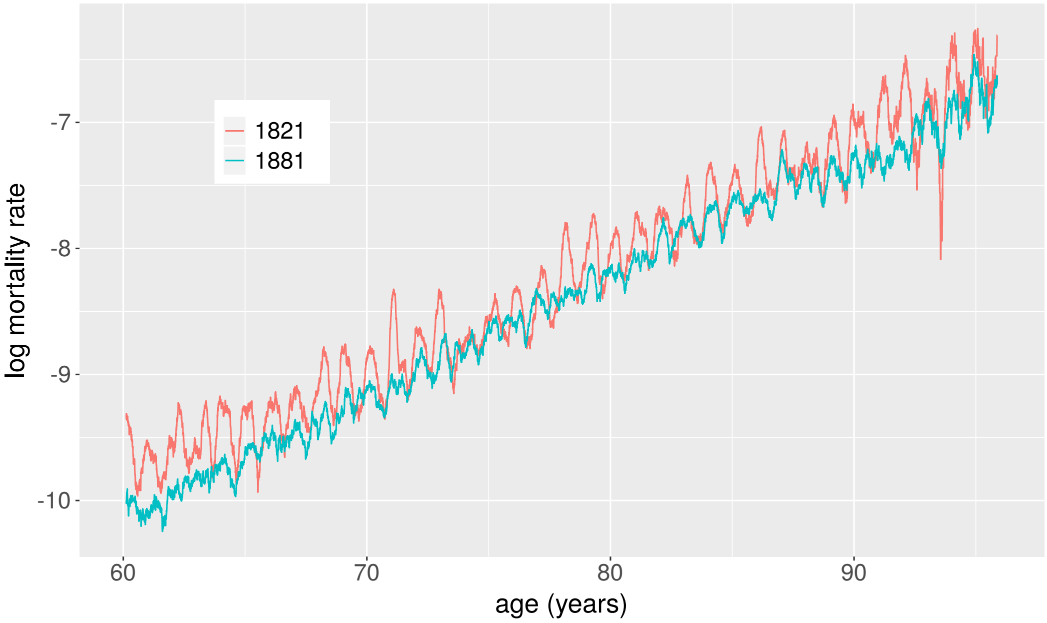

Figure S3: Mortality rates for two birth cohorts of men. Note how the annual troughs often coincide (and are sometimes even lower for the first cohort) but that peaks are consistently higher in the first cohort. This supports the decomposition of total mortality into lower-bound mortality and seasonal mortality. Life expectancy at 60 in the first cohort was 75.6 years compared to 77.2 in the last.

3 Tables

Birth year	# Men	# Women	Birth year	# Men	# Women
1800	10316	8933	1851	22489	19724
1801	11420	9551	1852	21407	18444
1802	12773	10769	1853	22744	19803
1803	12351	10411	1854	24903	21572
1804	12545	10651	1855	23331	20394
1805	12530	10652	1856	23452	20063
1806	12014	10442	1857	24362	20717
1807	12538	10729	1858	26313	22680
1808	11762	10077	1859	26741	23029
1809	11302	9254	1860	26585	22803
1810	13953	11870	1861	24930	21279
1811	15234	12854	1862	26251	22771
1812	14822	12480	1863	26806	23554
1813	13020	11005	1864	27289	23789
1814	14835	12589	1865	26681	23657
1815	16633	14226	1866	27056	24182
1816	17243	14674	1867	24706	21715
1817	16563	14218	1868	22269	19232
1818	17232	14940	1869	24069	21782
1819	16964	14831	1870	24803	22685
1820	17721	15405	1871	26511	24464
1821	19237	17040	1872	25821	24154
1822	20337	17375	1873	26920	24994
1823	21063	18616	1874	27345	25571
1824	20322	17450	1875	28234	27109
1825	21542	18654	1876	28501	27334
1826	20852	17887	1877	30084	28713
1827	18571	16238	1878	29555	27754
1828	20108	17220	1879	31155	29081
1829	20987	18256	1880	30322	28286
1830	20130	17342	1881	30411	28646
1831	18701	16318	1882	31120	28986
1832	19902	17466	1883	31825	29143
1833	22048	19303	1884	33288	31270
1834	22198	19253	1885	33584	31473
1835	21889	18925	1886	34618	32250
1836	21267	18061	1887	35420	32844
1837	20270	17602	1888	35422	32744
1838	19726	16761	1889	34697	31691
1839	20291	17042	1890	35793	32683
1840	21732	18788	1891	36838	33888
1841	20906	17883	1892	36219	33691
1842	22125	18723	1893	37396	34988
1843	21593	18591	1894	37994	35312
1844	22721	19759	1895	39235	37056
1845	21619	18499	1896	39465	37567
1846	20177	17533	1897	39756	38173
1847	20862	18025	1898	40954	38987
1848	22087	18700	1899	40727	38719
1849	23589	20301	1900	43108	40948
1850	22720	19583	1901	43583	42030

Table S1: Size of birth cohorts

Birth year	e_{59}	e_{59}^*	diff	Birth year	e_{59}	e_{59}^*	diff
1800	73.4	75.9	-2.6	1851	76.3	77.8	-1.6
1801	73.4	75.7	-2.3	1852	76.3	77.6	-1.4
1802	73.6	76.0	-2.4	1853	76.2	77.7	-1.5
1803	73.7	76.3	-2.6	1854	76.3	77.8	-1.4
1804	73.8	76.1	-2.3	1855	76.4	77.7	-1.4
1805	73.7	76.0	-2.2	1856	76.3	77.8	-1.5
1806	73.8	76.3	-2.5	1857	76.4	77.7	-1.3
1807	73.8	76.0	-2.2	1858	76.4	77.7	-1.3
1808	74.0	76.4	-2.5	1859	76.5	78.0	-1.5
1809	74.1	76.5	-2.4	1860	76.5	77.9	-1.3
1810	74.4	76.9	-2.5	1861	76.4	77.7	-1.3
1811	74.4	76.9	-2.4	1862	76.4	77.6	-1.2
1812	74.6	76.8	-2.2	1863	76.5	77.7	-1.2
1813	74.8	77.0	-2.3	1864	76.5	77.5	-1.1
1814	74.8	77.2	-2.3	1865	76.4	77.7	-1.2
1815	74.9	77.1	-2.2	1866	76.5	77.7	-1.2
1816	75.1	77.4	-2.3	1867	76.6	77.7	-1.1
1817	75.1	77.3	-2.2	1868	76.6	77.6	-1.1
1818	75.1	77.4	-2.3	1869	76.8	77.8	-1.0
1819	75.4	77.4	-2.0	1870	76.7	77.7	-1.0
1820	75.5	77.8	-2.3	1871	76.8	77.7	-0.9
1821	75.6	77.6	-2.1	1872	76.9	77.8	-1.0
1822	75.6	77.7	-2.1	1873	76.8	77.7	-0.9
1823	75.6	77.5	-2.0	1874	76.9	77.9	-1.0
1824	75.6	77.5	-1.9	1875	76.9	77.9	-0.9
1825	75.5	77.6	-2.1	1876	77.0	77.8	-0.9
1826	75.7	77.7	-1.9	1877	77.1	77.9	-0.8
1827	75.7	77.7	-2.0	1878	77.1	77.8	-0.7
1828	75.6	77.6	-2.0	1879	77.2	78.1	-0.9
1829	75.8	77.7	-1.9	1880	77.2	77.9	-0.7
1830	75.9	77.9	-2.0	1881	77.2	77.8	-0.6
1831	75.8	77.6	-1.8	1882	77.2	78.0	-0.8
1832	76.0	77.7	-1.7	1883	77.3	77.9	-0.7
1833	75.8	77.6	-1.8	1884	77.4	78.1	-0.6
1834	76.0	77.5	-1.6	1885	77.4	78.0	-0.6
1835	76.0	77.6	-1.6	1886	77.4	78.1	-0.6
1836	76.0	77.6	-1.6	1887	77.4	78.0	-0.6
1837	76.0	77.7	-1.6	1888	77.3	77.9	-0.6
1838	76.0	77.5	-1.6	1889	77.4	78.1	-0.7
1839	76.0	77.7	-1.6	1890	77.4	78.1	-0.7
1840	76.1	77.7	-1.6	1891	77.5	78.1	-0.6
1841	76.1	77.5	-1.4	1892	77.5	78.2	-0.7
1842	76.0	77.4	-1.4	1893	77.5	78.2	-0.7
1843	76.1	77.7	-1.7	1894	77.4	78.2	-0.7
1844	76.0	77.7	-1.7	1895	77.4	78.2	-0.7
1845	76.1	77.5	-1.4	1896	77.6	78.4	-0.8
1846	76.1	77.5	-1.4	1897	77.4	78.1	-0.7
1847	76.3	77.8	-1.6	1898	77.6	78.3	-0.7
1848	76.1	77.4	-1.3	1899	77.6	78.2	-0.7
1849	76.2	77.7	-1.5	1900	77.5	78.3	-0.8
1850	76.3	77.9	-1.6	1901	77.6	78.3	-0.6

Table S2: Survival data for men. e_{59} is the average life duration conditional upon reaching 59.5 years of age. e_{59}^* is the prediction from the model based on the lower-bound mortality, that is M_1 in Equation 4 in the main paper.

Birth year	e_{59}	e^*_{59}	diff	Birth year	e_{59}	e^*_{59}	diff
1800	74.8	78.2	-3.4	1851	77.2	79.2	-1.9
1801	75.0	78.0	-2.9	1852	77.2	79.0	-1.8
1802	75.0	78.0	-3.0	1853	77.1	78.9	-1.8
1803	75.2	78.6	-3.5	1854	77.2	79.1	-1.9
1804	75.3	78.7	-3.4	1855	77.1	78.9	-1.7
1805	75.1	78.1	-3.0	1856	77.2	78.8	-1.7
1806	75.2	78.6	-3.4	1857	77.2	78.8	-1.6
1807	75.3	78.7	-3.4	1858	77.1	78.7	-1.6
1808	75.4	78.7	-3.3	1859	77.2	78.8	-1.6
1809	75.5	78.9	-3.4	1860	77.1	78.7	-1.6
1810	75.8	79.0	-3.2	1861	77.2	78.7	-1.5
1811	75.8	78.9	-3.1	1862	77.3	78.9	-1.6
1812	75.9	79.0	-3.1	1863	77.3	78.6	-1.3
1813	75.9	78.9	-3.0	1864	77.3	78.8	-1.5
1814	76.1	79.4	-3.3	1865	77.4	78.8	-1.5
1815	76.2	79.4	-3.2	1866	77.4	78.7	-1.3
1816	76.2	79.3	-3.1	1867	77.3	78.6	-1.2
1817	76.1	79.2	-3.1	1868	77.5	78.8	-1.3
1818	76.2	79.0	-2.8	1869	77.4	78.7	-1.3
1819	76.4	79.1	-2.7	1870	77.6	78.7	-1.1
1820	76.4	79.3	-2.9	1871	77.6	78.7	-1.2
1821	76.6	79.2	-2.7	1872	77.8	78.9	-1.1
1822	76.5	79.5	-3.0	1873	77.7	78.8	-1.1
1823	76.7	79.6	-2.8	1874	77.8	78.9	-1.1
1824	76.6	79.4	-2.9	1875	78.0	78.9	-0.9
1825	76.5	79.4	-2.8	1876	78.0	79.1	-1.1
1826	76.7	79.3	-2.6	1877	78.1	79.2	-1.1
1827	76.8	79.4	-2.6	1878	78.3	79.3	-1.0
1828	76.8	79.3	-2.5	1879	78.4	79.3	-0.9
1829	76.8	79.1	-2.3	1880	78.5	79.5	-1.0
1830	76.9	79.5	-2.6	1881	78.6	79.4	-0.8
1831	77.0	79.4	-2.4	1882	78.6	79.5	-0.9
1832	76.9	79.4	-2.5	1883	78.8	79.6	-0.8
1833	76.9	79.2	-2.3	1884	79.1	79.9	-0.8
1834	77.0	79.1	-2.1	1885	79.0	80.0	-1.0
1835	77.2	79.3	-2.1	1886	79.3	80.1	-0.8
1836	77.1	79.3	-2.2	1887	79.4	80.2	-0.8
1837	77.1	79.3	-2.2	1888	79.4	80.0	-0.6
1838	77.0	79.3	-2.2	1889	79.7	80.5	-0.8
1839	77.1	79.5	-2.4	1890	79.8	80.5	-0.7
1840	77.2	79.3	-2.2	1891	80.1	80.8	-0.7
1841	77.1	79.2	-2.1	1892	80.3	81.2	-0.9
1842	77.0	79.0	-2.0	1893	80.4	81.2	-0.8
1843	77.1	79.1	-2.0	1894	80.5	81.4	-0.9
1844	77.1	79.0	-2.0	1895	80.6	81.4	-0.8
1845	77.1	78.9	-1.8	1896	80.8	81.5	-0.8
1846	77.0	78.8	-1.9	1897	80.9	81.7	-0.8
1847	77.1	79.1	-2.0	1898	81.1	81.9	-0.8
1848	77.1	79.1	-2.0	1899	81.3	82.1	-0.8
1849	77.2	79.2	-2.0	1900	81.5	82.3	-0.8
1850	77.1	78.8	-1.7	1901	81.7	82.4	-0.7

Table S3: Survival data for women. e_{59} is the average life duration conditional upon reaching 59.5 years of age. e^*_{59} is the prediction from the model based on the lower-bound mortality, that is M_1 in Equation 4 in the main paper.
